# Quality of life in patients following distal nerve transfers for the restoration of elbow flexion and extension- single center experience

**DOI:** 10.1016/j.bas.2026.106156

**Published:** 2026-07-02

**Authors:** Andrija Savić, Svetozar Stanković, Aleksandar Đurđević, Milan Lepić, Jovan Grujić, Aleksa Mićić, Nenad Novaković, Aleksandra Stojiljković, Lukas Rasulić

**Affiliations:** a Faculty of Medicine, University of Belgrade, Belgrade, Serbia; b Clinic for Neurosurgery, University Clinical Centre of Serbia, Belgrade, Serbia; c Medical Faculty, Military Medical Academy, University of Defence, Belgrade, Serbia; d Clinic for Neurosurgery, Military Medical Academy, Belgrade, Serbia; e Clinic for Neurosurgery, University Clinical Centre of Kragujevac, Kragujevac, Serbia

**Keywords:** Distal nerve transfer, Oberlin procedure, Thoracodorsal-to-triceps transfer, Brachial plexus injury, Elbow function restoration

## Abstract

**Introduction:**

Peripheral nerve injuries, though relatively uncommon, predominantly affect young adults after high-energy trauma and carry significant socioeconomic impact. Brachial plexus traction injuries are particularly challenging, with uncertain outcomes after conventional grafting. Distal nerve transfers shorten regeneration distance and avoid scarred zones, providing reliable restoration of elbow function.

**Research question:**

Can distal nerve transfers (Oberlin and thoracodorsal-to-triceps) reliably restore elbow flexion and extension and improve quality of life in patients with otherwise irreparable peripheral nerve injuries?

**Material and methods:**

We conducted a prospective study of 31 patients with upper extremity nerve injuries treated between 2016 and 2022. All underwent distal nerve transfers: Oberlin (n = 24), modified Oberlin (n = 2), thoracodorsal-to-triceps (n = 7). Outcomes were assessed at 24 months, including elbow flexion/extension, range of motion, supination, and quality of life using the PNSQoL score.

**Results:**

Functional recovery was achieved in >95% of patients. Among Oberlin procedures, 23/24 regained M3–M5 flexion, with 4 reaching M5; supination recovery paralleled flexion. Both modified Oberlin cases achieved M4–M5 strength. All thoracodorsal-to-triceps transfers restored functional extension (6 M5, 1 M4). Overall, mean PNSQoL score improved from 27.4 preoperatively to 66.5 postoperatively. Delayed surgery correlated negatively with strength and QOL recovery.

**Discussion and conclusion:**

Distal nerve transfers provide robust motor recovery and meaningful improvements in daily independence and social participation. The Oberlin transfer remains the benchmark for elbow flexion, while thoracodorsal-to-triceps transfer is a dependable option for extension. Early reconstruction maximizes outcomes. Future multicenter studies with standardized patient-reported outcomes are needed to further validate these findings.

## Highlights

• Distal nerve transfers restored elbow flexion and extension with >95% success.
• Oberlin transfer achieved reliable M3–M5 recovery in upper plexus injuries.
• Thoracodorsal-to-triceps transfer provided consistent triceps reinnervation.
• PNSQoL scores improved by 41 points, reflecting daily independence gains.
• Surgical delay strongly correlated with poorer motor and QOL outcomes.

## 1 Introduction

Loss of active elbow flexion represents the most functionally disabling deficit in adults, usually occurring as a part of complete or extended brachial plexus injuries (BPI), and it critically limits hand positioning and precludes most activities of daily living. Consequently, restoration of elbow flexion is universally regarded as the priority in adult brachial plexus reconstructive surgery, preceding shoulder abduction or hand reanimation (Chuang, 2010; Sinha et al., 2016; Terzis and Barbitsioti, 2012).

Elbow flexion and supination depend primarily on C5 fibers via the musculocutaneous nerve (biceps brachii, brachialis) and contributions from the radial nerve (brachioradialis, supinator), whereas elbow extension is mediated by the radial nerve to the triceps (predominantly C7–C8) (Johnson et al., 2010).

On this anatomical basis, recipient branches of the musculocutaneous and radial nerves are logical targets for reconstructing elbow flexion (Oberlin et al., 1994). Comparative studies have demonstrated that nerve transfers achieve faster and more reliable functional recovery than grafts, establishing them as the preferred reconstructive strategy for restoring elbow function in severe injuries (Flores, 2015; Garg et al., 2011; Merrell et al., 2001; Tung and Mackinnon, 2010).

Although functional recovery after nerve transfers has been well documented, three important aspects remain underexplored. First, triceps reinnervation procedures, particularly thoracodorsal-to-triceps transfer, are less frequently reported, despite their importance in elbow stability and upper-limb positioning. Second, the relationship between timing of surgery and long-term outcomes, though suggested in prior work, requires further prospective evaluation. Finally, most surgical series emphasize motor strength outcomes but rarely integrate patient-reported quality of life measures.

To address these gaps, we conducted a prospective observational study of 31 patients undergoing distal nerve transfers, primarily Oberlin procedure and thoracodorsal-to-triceps transfer. Our aims were to assess postoperative functional outcomes of elbow flexion, supination, and extension, evaluate quality of life using the PNSQoL instrument developed by our group (Savić et al., 2024, 2026; Rasulić et al., 2022) and examine the correlation between surgical timing and postoperative recovery.

## 2 Methods

### 2.1 Study design and patients

We conducted a prospective study of 31 patients with brachial plexus and peripheral nerve injuries of the upper extremity who underwent distal nerve transfer procedures at the Clinic for Neurosurgery, Clinical Center of Serbia between January 1, 2016, and December 31, 2022 (Appendix A). The study was conducted in accordance with the Declaration of Helsinki, and approved by the Institutional Ethics Committee of the Clinical Centre of Serbia, Belgrade, Serbia. Informed consent was obtained from all subjects involved in the study.

During the study period (January 2016 – December 2022), a total of 36 patients underwent distal nerve transfer procedures at our institution. Of these, 5 were excluded from the final analysis: 2 failed to complete the minimum 24-month follow-up (of whom 1 was lost during the COVID-19 pandemic period, 2020–2021), 2 did not adhere to the standardized postoperative physical therapy protocol, and 1 met other exclusion criteria (associated lower-extremity or cranial nerve injuries, or absence of informed consent). The remaining 31 patients constituted the final study cohort.

### 2.2 Inclusion criteria

- Age >18 years;
- Loss of elbow function due to peripheral nerve or brachial plexus injury;
- Underwent distal nerve transfer (Oberlin or thoracodorsal-to-triceps transfer);
- Compliance with standardized postoperative physical therapy and at least 24 months of follow-up.

### 2.3 Exclusion criteria

- Associated lower-extremity or cranial nerve injuries;
- Absence of informed consent;
- Failure to complete physical therapy or follow-up.

All patients were treated with at least one of the following distal nerve transfer procedures: the Oberlin transfer (transfer of a fascicle of the ulnar nerve to the branch of the musculocutaneous nerve for the biceps brachii) or the thoracodorsal to triceps transfer (transfer of a branch of the thoracodorsal nerve to the branch of the radial nerve for the long head of the triceps brachii).

Oberlin procedure was indicated in upper or extended brachial plexus injuries, or isolated proximal musculocutaneous nerve lesions, presenting with loss of active elbow flexion, in whom direct nerve repair or grafting was not feasible and adequate ulnar nerve donor function was preserved.

Patients are grouped in the analysis by surgical procedure performed rather than by primary diagnosis, which is consistent with the standard approach in nerve transfer series where eligibility is defined by the reconstructive target and donor availability.

Thoracodorsal-to-triceps transfer was indicated in patients with proximal radial nerve lesions or brachial plexus injuries involving the posterior cord, presenting with loss of active elbow extension, when direct reconstruction was not possible and preserved thoracodorsal nerve function was confirmed.

### 2.4 Preoperative evaluation

Baseline assessment included a detailed history, clinical and neurological examination, electrophysiological studies (electromyoneurography, somatosensory evoked potentials), and radiological imaging (ultrasound and magnetic resonance imaging).

### 2.5 Postoperative care and follow-up

All patients began standardized physical therapy three weeks after surgery, with a minimum duration of 12 weeks. Final outcome evaluation was performed 24 months postoperatively. No patient in our series reported clinically significant donor-site weakness.

### 2.6 Outcome measures

#### 2.6.1 Elbow flexion and forearm supination

For patients undergoing Oberlin procedure, outcomes included elbow flexion strength, elbow flexion range of motion (ROM), and forearm supination.

Strength was graded according to the Medical Research Council (MRC) Manual Muscle Testing (MMT) (M0–M5), with M0–M2 deemed unsatisfactory and M3–M5 satisfactory.

ROM was measured with a protractor, with angles recorded from full extension (180°) to full flexion (0°).

Supination was evaluated by observation with the forearm supported on a table and graded as no motion, slight, partial, or full motion.

#### 2.6.2 Elbow extension

For patients undergoing thoracodorsal-to-triceps transfer, outcomes included elbow extension strength and ROM.

Strength was assessed using the MRC MMT with M0–M2 deemed unsatisfactory and M3–M5 satisfactory.

ROM was measured using a protractor, with the upper arm in passive abduction and the forearm moving from full flexion to extension.

#### 2.6.3 Quality of life (QoL)

QOL was assessed using the PNSQoL (Peripheral Nerve Surgery Quality of Life) questionnaire (Appendix B). (Savić et al., 2024) The PNSQoL is a 16-item instrument developed by our group and adapted to the socioeconomic context of our patient population. It assesses functional independence (personal care, housework, shopping, occupational and recreational participation), psychosocial wellbeing, and satisfaction with extremity condition, social life, and professional life, yielding a continuous score of 0–80; all assessed domains are directly impacted by upper extremity nerve injury regardless of aetiology, justifying its application across both traumatic and neoplastic peripheral nerve surgery populations. The questionnaire was administered preoperatively and 24 months postoperatively, and results were compared.

### 2.7 Surgical procedures

#### 2.7.1 Oberlin transfer

**Fig. 1:**
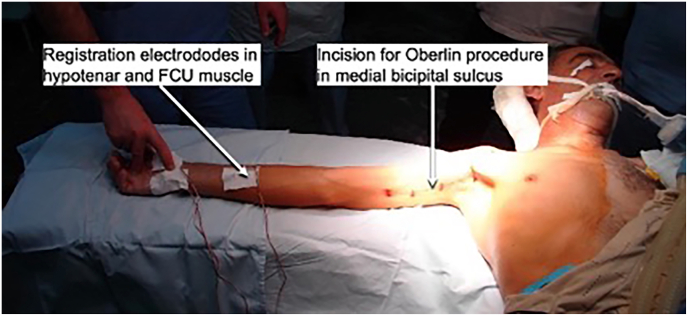
Patient positioning and surgical incision for exposure of the medial bicipital groove in preparation for the Oberlin procedure. Registration electrodes are placed in hypothenar and flexor carpi ulnaris (FCU) muscle**.**

**Fig. 2:**
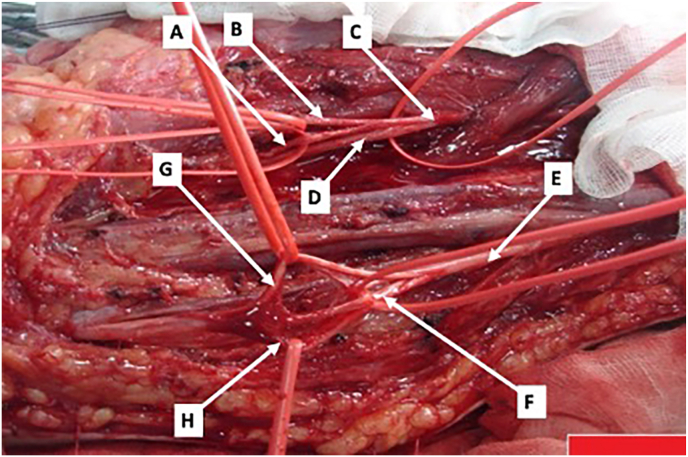
Intraoperative view showing the musculocutaneous nerve with its terminal branches and the ulnar nerve after neurolysis, during identification and harvesting of donor fascicles for the Oberlin transfer. **A-** Lateral cutaneal antebrachial nerve, **B**- biceps branch, **C**- musculocutaneous nerve, **D**-brachialis branch, **E**-ulnar nerve, **F-** second ulnar fascicular group, **G**-fascicular group for flexor carpi ulnaris muscle, **H-** third fasccular group.

**Fig. 3:**
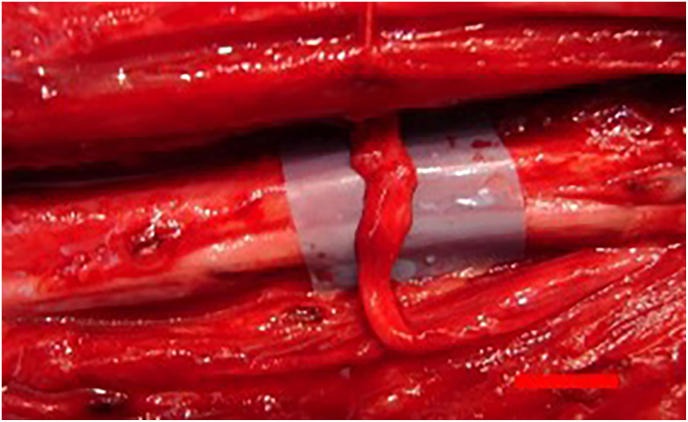
End-to-end microsurgical coaptation of the fascicular group for flexor carpi ulnaris to the branch of the musculocutaneous nerve for the biceps brachii, completing the Oberlin procedure.

Patients were positioned supine with the arm abducted (Fig. 1). Through a medial bicipital groove incision, the ulnar nerve was exposed and a fascicular group for the flexor carpi ulnaris was identified using intraoperative electrical stimulation. The musculocutaneous nerve was dissected between the biceps brachii and coracobrachialis, and the branch to the biceps was prepared as the recipient (Fig. 2). The donor fascicle of the ulnar nerve was sectioned distally, and the recipient branch was cut proximally to allow a tension-free end-to-end coaptation under the operating microscope (Fig. 3). In all cases (including other techniques), sutures were placed and reinforced with fibrin glue.

In selected cases where no graftable proximal musculocutaneous stump was available (iatrogenic injuries), a *Modified Oberlin procedure* was performed. This technique combined transfer of a fascicle of the median nerve to the branch of the musculocutaneous nerve for the biceps brachii, together with transfer of a fascicle of the ulnar nerve to the branch for the brachialis muscle.

#### 2.7.2 Thoracodorsal-to-triceps transfer

**Fig. 4:**
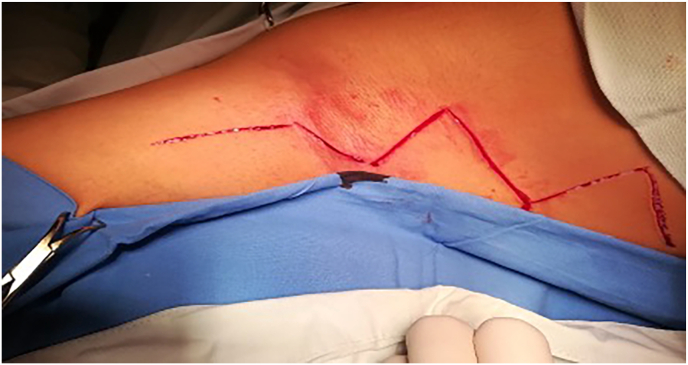
Incision at the level of the right axilla and the lateral side of the ribcage for exposure of the neurovascular bundle and identification of the thoracodorsal nerve, as the first step of the thoracodorsal-to-triceps transfer.

**Fig. 5:**
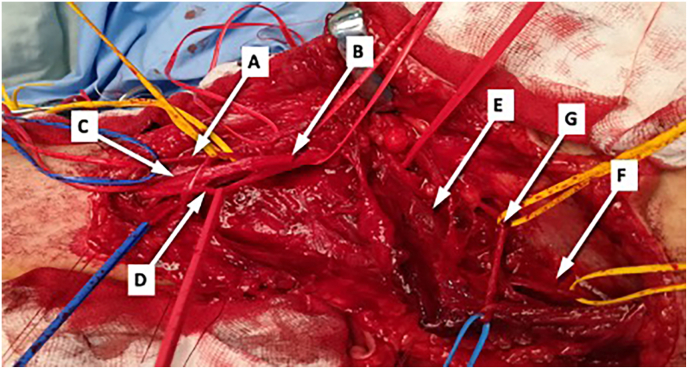
Intraoperative dissection showing the radial nerve with its branch for the long head of the triceps brachii muscle and the thoracodorsal nerve with its two terminal branches, during preparation for the thoracodorsal-to-triceps transfer. **A**-Branch for lateral head of triceps muscle, **B-**radial nerve, **C**-branch for medial head of triceps muscle, **D**-branch for long head of triceps muscle, **E**-thoracodorsal nerve, **G**-terminal branch for lateral part of latissimus dorsi muscle, **F**- terminal branch for medial part of latissimus dorsi muscle.

**Fig. 6:**
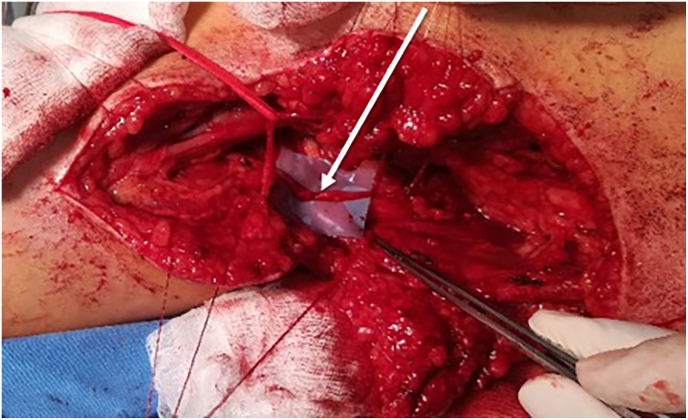
Direct end-to-end coaptation between the donor branch of the thoracodorsal nerve (branch for medial part of latissimus dorsi muscle) and the recipient branch of the radial nerve for the long head of the triceps muscle, completing the thoracodorsal-to-triceps nerve transfer.

With the patient supine and the arm hyperabducted, a linear axillary–medial arm incision was made to expose the thoracodorsal nerve on the deep surface of the latissimus dorsi (Fig. 4). One of its terminal branches was selected as the donor to preserve partial latissimus function. The radial nerve was then identified above the tendon of the latissimus dorsi, and the branch to the long head of the triceps was prepared as the recipient (Fig. 5). Donor and recipient nerves were divided and coapted end-to-end without tension (see Fig. 6)**.**

In patients with upper or extended upper brachial plexus injuries, shoulder stability and abduction/external rotation were assessed as part of preoperative planning. When indicated, elbow reconstruction was combined with shoulder-directed procedures, including spinal accessory-to-suprascapular nerve transfer and/or axillary nerve reconstruction.

In patients with supra- or infraclavicular brachial plexus injuries, the proximal lesion was evaluated intraoperatively when indicated; distal nerve transfer was selected when proximal reconstruction was not feasible, was unlikely to reinnervate the target in time, or was used as part of a combined reconstruction strategy. Root avulsion was confirmed preoperatively by MRI and electrophysiological studies precluding proximal repair. In patients with isolated proximal radial nerve injuries, extensive perineural scarring and proximity to major vascular structures made proximal exploration hazardous, and distal transfer was therefore the preferred reconstructive strategy.

#### 2.7.3 Statistical analysis

Data were analyzed using IBM SPSS Statistics for Windows, Version 22.0 (IBM Corp., Armonk, NY, USA). Descriptive statistics were used to summarize patient demographics, injury characteristics, and functional outcomes. Continuous variables are presented as means with ranges, while categorical variables are reported as frequencies and percentages. Comparative analyses of preoperative and postoperative results were performed where appropriate. A *p*-value <0.05 was considered statistically significant.

## 3 Results

A total of 31 patients were included in the study, with a mean age of 33.6 years (range 18–61). The cohort was predominantly male (87.1%, n = 27). The majority of injuries resulted from traffic accidents (64.5%, n = 20), followed by iatrogenic injuries (16.1%, n = 5) and other mechanisms including cuts (n = 3), falls from height (n = 2), and an object falling on the patient (n = 1). Upper brachial plexus palsy was the most frequent nerve lesion type (71.0%, n = 22), followed by isolated radial nerve lesions (9.7%, n = 3), isolated musculocutaneous nerve lesions (6.5%, n = 2), and extended upper brachial plexus palsy (3.2%, n = 1); infraclavicular lesions with predominant radial nerve involvement accounted for the remainder (ICLR n = 1, ICLRM n = 1, ICLRU n = 1; 9.7% combined). Associated injuries, most often fractures and vascular trauma, were present in 64.5% of patients (n = 20). The mean interval between injury and nerve transfer was 6.7 months (range 1–15)

Regarding surgical procedures, the triple nerve transfer (TNT: spinal accessory-to-suprascapular, long head of triceps branch-to-axillary, and Oberlin transfer) was the most commonly performed, applied in 22 patients (71.0%) with upper brachial plexus palsy. Two patients with iatrogenic isolated musculocutaneous nerve lesions underwent a modified Oberlin procedure. Seven patients (22.6%) underwent thoracodorsal-to-triceps transfer as part of their reconstruction, including two patients in whom it was combined with an Oberlin transfer to address both elbow flexion and extension. Table 1 summarizes all patients according to target elbow function (flexion, extension, or both), the specific reconstructive procedure performed, and nerve lesion type, and presents functional and quality-of-life outcomes for each group.

**Table 1:** Functional and quality-of-life outcomes according to the target elbow function, reconstructive procedure and nerve lesion type.

Target function	Surgical procedure(s)	Nerve lesion type	N	Elbow flexion strength	Elbow extension strength	Satisfactory outcome^a^	Full Range of Motion	Preoperative PNSQoL, Mean (Range)	Postoperative PNSQoL, Mean (Range)	ΔPNSQoL
**Elbow flexion**	TNT (SA-SS + LHT-A + O)	UP	22	M5 = 3, M4 = 12, M3 = 6, M2 = 1	/	21 (95.5%)	21 (95.5%)	22.5 (15–31)	62.9 (31–80)	+40.4
	mO	ILMC	2	M5 = 1, M4 = 1	/	2 (100%)	2 (100%)	39.0 (36–42)	76.5 (73–80)	+37.5
**Elbow flexion & extension**^b^	PM-A + TD-LHT + SA-SS + O	EUP	1	M4 = 1	M4 = 1	1 (100%)	1 (100%)	18	55	+37
	TD-LHT + FCR-IP + FDS-ECRB + O	ICLRM	1	M5 = 1	M5 = 1	1 (100%)	1 (100%)	27	76	+49
	Overall (O + TD-LHT)		2	M5 = 1, M4 = 1	M5 = 1, M4 = 1	2 (100%)	2 (100%)	22.5 (18–27)	65.5 (55–76)	+43.0
**Elbow extension**	TD-LHT + FCR-IP + FDS-ECRB	ICLR	1	/	M5 = 1	1 (100%)	1 (100%)	52	80	+28
	TD-LHT + FCR-IP + FDS-ECRB + PQ-MFU + LCN-SFU	ICLRU	1	/	M5 = 1	1 (100%)	1 (100%)	39	78	+39
	GR + TD-LHT + FCR-IP + FDS-ECRB + PT-ECRB	ILR	1	/	M5 = 1	1 (100%)	1 (100%)	49	80	+31
	GR + TD-LHT + FCR-IP + FDS-ECRB	ILR	2	/	M5 = 2	2 (100%)	2 (100%)	46.5 (45–48)	79.0 (78–80)	+32.5
**Elbow flexion**	Overall (O + mO)		26	M5 = 5, M4 = 13, M3 = 7, M2 = 1	/	25 (96.2%)	25 (96.2%)	23.7 (15–42)	64.1 (31–80)	+40.4
**Elbow extension**	Overall (TD-LHT)		7	/	M5 = 6, M4 = 1	7 (100%)	7 (100%)	39.7 (18–52)	75.3 (55–80)	+35.6
**All**	All		31	/	/	30 (96.8%)	30 (96.8%)	27.4 (15–52)	66.5 (31–80)	+39.1

**Abbreviations: Surgical procedure(s)**: TNT - triple nerve transfer: SA-SS - spinal accessory nerve to suprascapular nerve transfer, LHT-A - long head of triceps branch to axillary nerve transfer, O - Oberlin transfer; mO - modified Oberlin transfer; PM-A - medial pectoral nerve to axillary nerve transfer; TD-LHT - thoracodorsal nerve to triceps branch transfer; FCR-IP - flexor carpi radialis branch to posterior interosseous nerve transfer; FDS-ECRB - flexor digitorum superficialis branch to extensor carpi radialis brevis branch transfer; PQ-MFU - anterior interosseous nerve terminal branch to motor fascicle of the ulnar nerve transfer; LCN-SFU - lateral cutaneous nerve of the forearm to sensory fascicle of the ulnar nerve transfer; PT-ECRB - pronator teres tendon to extensor carpi radialis brevis tendon transfer; GR - graft repair; Nerve lesion type: UP - upper brachial plexus palsy; EUP - extended upper brachial plexus palsy; ICLR - infraclavicular brachial plexus lesion with predominant radial nerve involvement; ICLRM - infraclavicular brachial plexus lesion with predominant radial and musculocutaneous nerve involvement; ICLRU - infraclavicular brachial plexus lesion with predominant radial and ulnar nerve involvement; ILMC - isolated musculocutaneous nerve lesion; ILR - isolated radial nerve lesion.

a Satisfactory outcome defined as M3–M5 muscle strength according to the Medical Research Council (MRC) grading scale.

b Patients undergoing reconstruction of both elbow flexion and elbow extension contributed to both functional outcome analyses.

Functional recovery was generally excellent (Table 1). Among patients treated with the Oberlin transfer, elbow flexion of at least M3 strength was achieved in 95.8% (23/24), with five patients reaching M5 strength. Supination recovery paralleled flexion outcomes, with satisfactory function in 23 patients. The single unsatisfactory case involved a patient who presented 15 months after injury and underwent delayed reconstruction. Modified Oberlin procedures yielded one M5 and one M4 result, both satisfactory.

For thoracodorsal-to-triceps transfers, all seven patients regained functional extension (M3–M5), including six with full M5 strength. Elbow range of motion was fully restored in all groups.

Multivariable linear regression analysis was performed to identify factors associated with postoperative muscle strength and quality-of-life outcomes. Covariates included age, sex, injury type, nerve lesion type, and time between injury and reconstructive surgery. For postoperative muscle strength, a longer interval between injury and surgery was independently associated with lower postoperative MRC grade (β = −0.184, p = 0.002). No significant associations were observed for age, sex, injury type, or nerve lesion type. For postoperative PNSQoL, a longer interval between injury and surgery was independently associated with worse postoperative quality-of-life scores (β = −3.39, p < 0.001). Nerve lesion type was also associated with postoperative PNSQoL, with patients with ICLRU lesions demonstrating higher postoperative scores than the reference category (β = 24.1, p = 0.022). No significant associations were observed for age, sex, or injury type.

Of the 24 patients in the classic Oberlin subgroup, 22 had upper brachial plexus palsy (UP, C5–C6); one patient with extended upper brachial plexus palsy (EUP, C5–C7) and one with an infraclavicular lesion (ICLRM) underwent Oberlin transfer as part of combined multi-procedure reconstructions. The lesion type covariate therefore reflects the distinction between UP and non-UP indications within this subgroup, and its non-significant result (p = 0.674) is consistent with the predominantly homogeneous lesion composition.

**Fig. 7:**
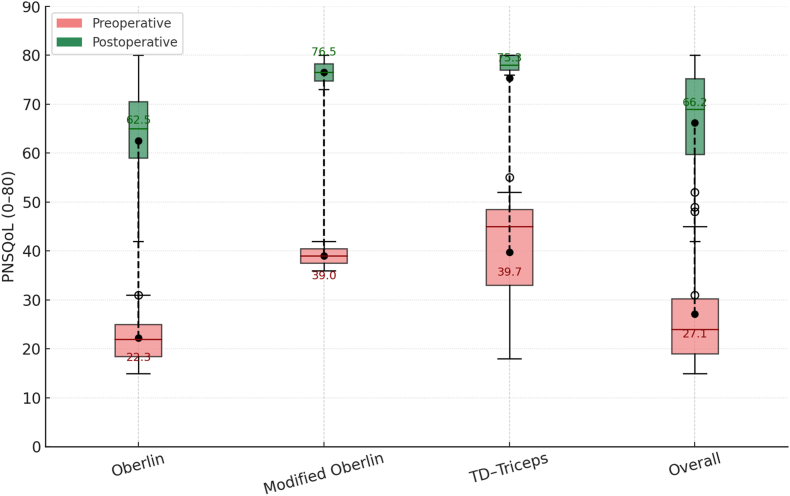
Preoperative vs postoperative PNSQoL Scores by Surgery Group.

**Fig. 8:**
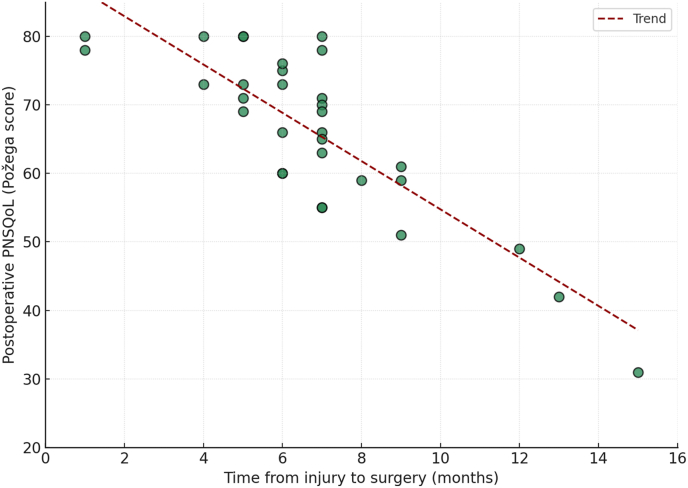
Correlation between time from injury to surgery and postoperative quality of life (PNSQoL score). A strong negative correlation was observed (Pearson's r = −0.84, p < 0.001), indicating that longer surgical delay was associated with lower postoperative quality of life.

**Fig. 9:**
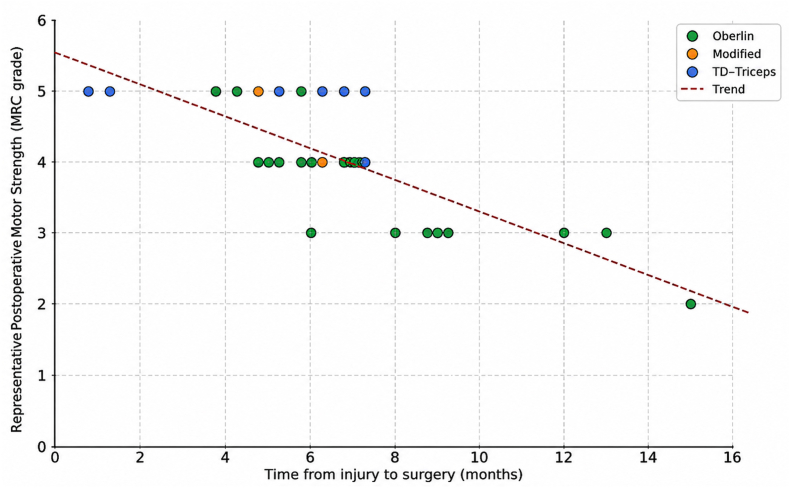
Correlation between time from injury to surgery and postoperative muscle strength recovery (MRC grade). A strong negative correlation was observed (Pearson's r = −0.78, p < 0.001), indicating that longer surgical delay was associated with reduced postoperative motor strength.

Quality of life, measured by the PNSQoL score, improved markedly across all surgical groups (Table 1). The mean overall score increased from 27.4 (range 15–52) preoperatively to 66.5 (range 31–80) postoperatively, corresponding to an average improvement of 39.1 points (p < 0.001). Similar improvements were observed in subgroup analyses, with the largest postoperative values in patients undergoing modified Oberlin transfer. Fig. 7 illustrates upward shift in PNSQoL scores across all groups. Fig. 9 demonstrates a negative correlation between the time elapsed to surgery and postoperative muscle strength recovery (see Fig. 8).

Multivariable linear regression (covariates: age, sex, injury type, nerve lesion type, time to surgery) identified longer surgical delay as the only independent predictor of both lower postoperative MRC grade (β = −0.184, p = 0.002) and worse postoperative PNSQoL score (β = −3.39, p < 0.001), with no significant associations observed for any other covariate.

## 4 Discussion

Restoration of elbow flexion and extension remains the cornerstone of functional recovery after brachial plexus and major peripheral nerve injuries. Despite ongoing debate about donor selection and timing (Belzberg et al., 2004), our series shows that distal nerve transfers consistently restored function, with >95% of patients reaching at least M3 strength and reporting substantial QOL gains. These findings are consistent with the accumulated global experience, which now positions distal transfers as the preferred reconstructive strategy in adults (Bertelli et al., 2024; Moucharafieh et al., 2020; Moore, 2014).

Oberlin procedure has become the benchmark since its original description in 1994 (Oberlin et al., 1994). Teboul et al. achieved satisfactory elbow flexion in 92% of 32 patients (Teboul et al., 2004), while Mackinnon and colleagues demonstrated that single fascicular ulnar-to-biceps transfer reliably restores M4–M5 strength in most cases (Mackinnon et al., 2005; Novak et al., 2002). Recent series further confirm recovery rates exceeding 90%, particularly when surgery is performed within 6 months of injury (Maldonado et al., 2017; Gohritz et al., 2023). In line with these data, our patients achieved a 96.2% rate of satisfactory recovery, comparable to recent multicenter analyses (Bertelli et al., 2024; Maldonado et al., 2017), underscoring that a single fascicular transfer provides sufficient donor axons for effective biceps reinnervation. Although double fascicular techniques have been proposed to enhance brachialis reinnervation (Novak et al., 2002; Shekouhi et al., 2024), comparative reviews have not demonstrated consistent superiority over the classic Oberlin procedure and suggested a higher risk of donor morbidity (Sneiders et al., 2019). Notably, in our series, two iatrogenic cases required modified Oberlin transfers, both resulting in M4–M5 recovery, supporting their use in selected patients.

Functional triceps recovery is essential for elbow stability and upper limb positioning. Although less frequently explored in the literature, thoracodorsal-to-triceps transfer has emerged as a safe and effective solution for proximal radial nerve lesions (Soldado et al., 2016). In our patients, 100% achieved functional extension, with 6 of 7 reaching M5 strength, in line with results from Soldado et al. (2016) and recent systematic review (Yang et al., 2012). Concerns about sacrificing part of latissimus dorsi function appear to be clinically minor, particularly when only one branch is harvested, while the donor provides an excellent size and axon match to the triceps branch (Lee and Mun, 2014).

Early reconstruction remains critical. Consistent with prior studies (Garg et al., 2011; Samardzić et al., 2012) the only unsatisfactory flexion outcome occurred in a patient operated 15 months after a high-energy traumatic upper plexus injury. Although surgical delay was the most apparent adverse factor, patient age and injury severity may also have contributed. Nevertheless, even delayed transfers can provide partial recovery, underscoring the advantage of distal coaptation near target muscles. Importantly, our study extends beyond traditional motor outcomes by documenting significant improvements in patient-reported QOL. A mean gain of 39.1 points in the PNSQoL score highlights that restored elbow function translates directly into independence in daily living and social participation, an aspect underrepresented in most surgical series (Allio et al., 2025; Dolan et al., 2012).

For the Oberlin transfer, only a single fascicle to the flexor carpi ulnaris was harvested from the ulnar nerve under intraoperative electrical stimulation guidance, leaving all remaining ulnar nerve function intact; this selective harvest is precisely why the technique is considered exceptionally safe and has been widely adopted. For the thoracodorsal-to-triceps transfer, only the medial terminal branch of the thoracodorsal nerve was used as donor, preserving the lateral branch and partial latissimus dorsi innervation; residual functional compromise is negligible, as shoulder adduction is additionally served by the pectoralis major, teres major, and subscapularis. No patient in our series reported clinically significant donor-site weakness, consistent with published morbidity data (Lee and Mun, 2014).

From a clinical standpoint, our findings support early adoption of distal nerve transfers as a primary reconstructive strategy in adult brachial plexus and proximal peripheral nerve injuries when direct repair or grafting is not feasible. The high and consistent recovery rates observed with the Oberlin procedure reinforce its role as the standard option for restoration of elbow flexion, while the excellent outcomes achieved with thoracodorsal-to-triceps transfer emphasize the importance of actively addressing elbow extension to ensure joint stability and effective upper-limb positioning. Importantly, the strong correlation between surgical delay and both motor and quality-of-life outcomes underscores the need for early referral to specialized centers. Together, these results suggest that comprehensive reconstruction of elbow flexion and extension, combined with timely intervention, has a direct and meaningful impact on patient independence and daily functioning.

When we categorize PNSQoL scores (total 0–80; poor 0–39, fair 40–49, good 50–59, very good 60–69, excellent 70–80), all patients undergoing the Oberlin transfer (n = 24) had poor preoperative QOL (24/24, 100%; mean PRP 22.1), but improved postoperatively to predominantly very good/excellent levels (16/24, 66.7%), with a mean POP of 62.2 and a mean gain of +40.1 points. In the modified Oberlin subgroup (n = 2), preoperative scores were poor/fair (mean PRP 39.0), yet both patients reached excellent postoperative QOL (2/2, 100%; mean POP 76.5; mean gain +37.5). For thoracodorsal-to-triceps transfer (n = 7), baseline QOL was mainly poor/fair (6/7, 85.7%; mean PRP 39.7), whereas postoperative outcomes clustered at the excellent level (6/7, 85.7%; mean POP 75.3), corresponding to a mean improvement of +35.6 points, indicating that restoration of elbow extension translated into substantial patient-perceived functional and social benefit.

Although 24 months was selected as the evaluation endpoint to ensure complete motor reinnervation and full rehabilitation benefit prior to assessment, serial functional and quality-of-life evaluations at earlier time points were not pre-specified, limiting our ability to characterise the trajectory of recovery, and represent an important consideration for future prospective series.

This is a single-center series with a statistically modest sample size and without a control group, limiting broader generalization. As a descriptive observational study, it is not designed to provide comparative effectiveness between techniques, but rather to present institutional outcomes and demonstrate the consistency and clinical relevance of distal nerve transfer strategies. The observed recovery rates were high; however, the precision of these estimates was limited by our sample size, indicating that larger cohorts are required to narrow confidence intervals around these effect estimates. The majority of procedures were performed in relatively young, otherwise healthy trauma patients which might pose a bias. Additionally, the study relies on a disease-specific patient-reported outcome measure (PNSQoL) developed by our group, which, although previously applied and clinically intuitive, has not yet undergone extensive external validation or direct comparison with widely used instruments, therefore, quality-of-life findings should be interpreted with caution. Nonetheless, the consistency of our results with multicenter cohorts supports the external validity of our findings.

## 5 Conclusion

Distal nerve transfers for elbow function restoration - Oberlin transfer for flexion and thoracodorsal-to-triceps transfer for extension, provided reliable restoration of elbow function in our series. Satisfactory motor recovery (≥M3) was achieved in more than 95% of patients, with 95.8% regaining functional elbow flexion and 100% achieving functional elbow extension. Elbow range of motion was fully restored across all groups, and forearm supination recovery closely paralleled flexion outcomes. These functional gains translated into marked improvements in patient-reported quality of life, with PNSQoL scores improving from a mean of 27.4 preoperatively to 66.5 postoperatively, shifting from predominantly poor/fair to mainly very good or excellent categories. Despite these consistent outcomes, the evidence base in peripheral nerve surgery remains limited to single-center series, and meaningful improvement can only be achieved through multicentric collaboration and prospective study designs.

## Declaration of competing interest

The authors declare that they have no known competing financial interests or personal relationships that could have appeared to influence the work reported in this paper.
