# Supplementary material for: Quality of life in patients following distal nerve transfers for the restoration of elbow flexion and extension- single center experience
Source: Brain Spine. 2026 Jul 2;6:106156. doi: 10.1016/j.bas.2026.106156 (PMC13348191; doi:10.1016/j.bas.2026.106156)
Supplement: Multimedia component 1 [file mmc1.docx]

***Appendix A.*** Patients, injuries, and procedures characteristics with outcomes

| **Number** | **Age** | **Gender** | **Type of injury** | **Nerve lesion type** | **Associated injuries** | **Emergency surgeries** | **Time between injury and surgery** | **Surgical procedure(s)** | **Results** |
| --- | --- | --- | --- | --- | --- | --- | --- | --- | --- |
| 1 | 23 | M | TAFSP | UP | CC | / | 4 | TNT | EF: M5, FRoM  SU: FRoM  PrePNSQoL: 19  PostPNSQoL:80 |
| 2 | 61 | M | TAD | UP | FH,  FV | OH,  IC | 9 | TNT | EF: M3, FRoM  SU: FRoM  PrePNSQoL: 25  PostPNSQoL:61 |
| 3 | 49 | M | C | UP | BVL | RV | 5 | TNT | EF: M4, FRoM  SU: FRoM  PrePNSQoL: 25  PostPNSQoL:73 |
| 4 | 28 | M | MTA | UP | FCS,  FF | TD,  OF | 6 | TNT | EF: M4, FRoM  SU: FRoM  PrePNSQoL: 23  PostPNSQoL:66 |
| 5 | 35 | M | C | UP | BVL | RV | 7 | TNT | EF: M4, FRoM  SU: FRoM  PrePNSQoL: 30  PostPNSQoL:71 |
| 6 | 51 | F | C | UP | BVL | RV | 8 | TNT | EF: M3, FRoM  SU: FRoM  PrePNSQoL: 28  PostPNSQoL:59 |
| 7 | 39 | M | MTA | UP | / | / | 6 | TNT | EF: M5, FRoM  SU: FRoM  PrePNSQoL: 25  PostPNSQoL:75 |
| 8 | 26 | M | MTA | UP | / | / | 5 | TNT | EF: M4, FRoM  SU: FRoM  PrePNSQoL: 18  PostPNSQoL:71 |
| 9 | 28 | M | MTA | UP | / | / | 7 | TNT | EF: M4, FRoM  SU: FRoM  PrePNSQoL: 20  PostPNSQoL:66 |
| 10 | 45 | M | MTA | UP | FT,  FR,  FMT,  FZ,  FM,  SF,  FV,  CTC | OT,  OR,  OMT,  OZ,  OM | 9 | TNT | EF: M3, FRoM  SU: FRoM  PrePNSQoL: 15  PostPNSQoL:51 |
| 11 | 33 | M | MTA | UP | FS,  FC,  FV | / | 7 | TNT | EF: M4, FRoM  SU: FRoM  PrePNSQoL: 29  PostPNSQoL:70 |
| 12 | 27 | M | OF | UP | SF,  FC,  HED,  FV,  LL | S | 9 | TNT | EF: M3, FRoM  SU: FRoM  PrePNSQoL: 22  PostPNSQoL:59 |
| 13 | 18 | F | MTA | UP | FC | / | 5 | TNT | EF: M4, FRoM  SU: FRoM  PrePNSQoL: 17  PostPNSQoL:69 |
| 14 | 30 | M | TAD | UP | FC,  FH | OH | 6 | TNT | EF: M3, FRoM  SU: FRoM  PrePNSQoL: 23  PostPNSQoL:60 |
| 15 | 35 | M | TAFSP | UP | / | / | 7 | TNT | EF: M4, FRoM  SU: FRoM  PrePNSQoL: 20  PostPNSQoL:65 |
| 16 | 33 | M | MTA | UP | / | / | 4 | TNT | EF: M5, FRoM  SU: FRoM  PrePNSQoL: 22  PostPNSQoL:73 |
| 17 | 22 | M | HF | UP | FC | / | 7 | TNT | EF: M4, FRoM  SU: FRoM  PrePNSQoL: 31  PostPNSQoL:63 |
| 18 | 31 | M | TAFSP | UP | FR,  FU,  CC | OR,  OU | 6 | TNT | EF: M4, FRoM  SU: FRoM  PrePNSQoL: 24  PostPNSQoL:60 |
| 19 | 49 | M | MTA | UP | CTC,  FV,  FCS,  LL | TD,  S | 13 | TNT | EF: M3, FRoM  SU: FRoM  PrePNSQoL: 19  PostPNSQoL:42 |
| 20 | 32 | M | TAFSP | UP | FR,  FMC,  FT | OT,  OR | 7 | TNT | EF: M4, FRoM  SU: FRoM  PrePNSQoL: 18  PostPNSQoL:69 |
| 21 | 46 | M | MTA | UP | CTC,  FC,  FH,  FV,  FCS,  LL | S,  OH | 15 | TNT | EF: M2, RoM: 0°  SU: RoM 0°  PrePNSQoL: 24  PostPNSQoL:31 |
| 22 | 33 | M | TAD | UP | / | / | 12 | TNT | EF: M3, FRoM  SU: FRoM  PrePNSQoL: 17  PostPNSQoL:49 |
| 23 | 30 | M | TAD | EUP | FH,  FC | OH | 7 | PM-A,  TD-LHT,  SA-SS,  O | EF: M4, FRoM  SU: FRoM  EX: M4, FRoM  PrePNSQoL: 18  PostPNSQoL:55 |
| 24 | 26 | M | MTA | ICLR | FH | OH | 5 | TD-LHT,  FCR-IP,  FDS-ECRB | EX: M5, FRoM  PrePNSQoL: 52  PostPNSQoL:80 |
| 25 | 35 | M | HF | ICLRM | FS,  FCS | / | 6 | TD-LHT,  FCR-IP,  FDS-ECRB,  O | EF: M5, FRoM  SU: FRoM  EX: M5, FRoM  PrePNSQoL: 27  PostPNSQoL:76 |
| 26 | 18 | M | TAD | ICLRU | FMT,  FM,  CTC,  CL | SC | 7 | TD-LHT,  FCR-IP,  FDS-ECRB,  PQ-MFU  LCN-SFU | EX: M5, FRoM  PrePNSQoL:39  PostPNSQoL:78 |
| 27 | 27 | M | IA | ILMC | / | / | 5 | mO | EF: M5, FRoM  SU: FRoM  PrePNSQoL: 42  PostPNSQoL:80 |
| 28 | 25 | M | IA | ILMC | / | / | 6 | mO | EF: M4, FRoM  SU: FRoM  PrePNSQoL: 36  PostPNSQoL:73 |
| 29 | 48 | F | IA | ILR | / | / | 7 | GR,  TD-LHT,  FCR-IP,  FDS-ECRB,  PT-ECRB | EX: M5, FRoM  PrePNSQoL: 49  PostPNSQoL:80 |
| 30 | 37 | M | IA | ILR | / | / | 1 | GR,  TD-LHT,  FCR-IP,  FDS-ECRB | EX: M5, FRoM  PrePNSQoL:48  PostPNSQoL:78 |
| 31 | 23 | F | IA | ILR | / | / | 1 | GR,  TD-LHT,  FCR-IP,  FDS-ECRB | EX: M5, FRoM  PrePNSQoL: 45  PostPNSQoL:80 |

**Abbreviations:** ***Gender:*** M - male, F - female; ***Type of injury:*** TAFSP - traffic accident with the front seat passenger in the car, TAD - traffic accident with the driver in the car, MTA - motorcycle traffic accident, C - cut, HF - fall from height, OF - an object fell on the patient, IA - iatrogenic injury; ***Nerve lesion type:*** UP - upper brachial plexus palsy, EUP - extended upper brachial plexus palsy, ICLR-infraclavicular brachial plexus lesion with a predominant affection of the radial nerve, ICLRM - infraclavicular brachial plexus lesion with a predominant affection of the radial nerve and the musculocutaneous nerve, ICLRU - infraclavicular brachial plexus lesion with a predominant affection of the radial nerve and the ulnar nerve, ILMC - isolated lesion of the musculocutaneous nerve, ILR - isolated lesion of the radial nerve; ***Associated injuries:*** CC - commotio cerebri, FH - humerus fracture, FC - fracture of the clavicle, FV - fracture of a vertebra, CTC - cerebral contusion, BVL - blood vessel lesion, FCS - ribs fracture, FF - fracture of the femur, LL - spleen lesion, FR - fracture of the radius, FMT - fracture of the metatarsal bone, FZ - fracture of the zygomatic bone, FM - - fracture of the mandible, SF - skull fracture, FS - fracture of the scapula, HED - epidural hematoma, FU-fracture of the ulna, FMC-fracture of the metacarpal bone, CL - colon lesion; ***Emergency surgeries:*** OH - humerus osteosynthesis, IC-instrumentalization of the spine, RV-blood vessel reconstruction, TD - thoracic drainage, OF - femur osteosynthesis, OT - tibia osteosynthesis, S-splenectomy, OR - radius osteosynthesis, OMT - metatarsal bone osteosynthesis, OZ - zygomatic bone osteosynthesis, OM - mandible osteosynthesis, OU - ulna osteosynthesis, SC - colon suture; ***Surgical procedure(s):*** TNT - “triple nerve transfer” - the transfer of the spinal accessory nerve to the suprascapular nerve, transfer of the branch of the radial nerve for the medial head of the triceps muscle to the branches of the axillary nerve for the anterior division of the deltoideus muscle and the teres minor muscle, transfer of fascicle of the ulnar nerve for the flexor carpi ulnaris muscle to the branch of the musculocutaneous nerve for the biceps brachii muscle, PM-A - the transfer of the medial pectoral nerve to the axillary nerve, SA-SS - the transfer of the spinal accessory nerve to the suprascapular nerve, O - Oberlin’s procedure (the transfer of the fascicle of the ulnar nerve for the flexor carpi ulnaris muscle to the branch of the musculocutaneous nerve for the biceps brachii muscle), mO - modified Oberlin’s procedure (the transfer of the fascicle of the median nerve for the flexor carpi radialis muscle to the branch of the musculocutaneous nerve for the biceps brachii muscle, and the transfer of the fascicle of the ulnar nerve for the flexor carpi ulnaris muscle to the branch of the musculocutaneous nerve for the brachialis muscle), TD-LHT - the transfer of the branch of the thoracodorsal nerve to the branch of the radial nerve for the long head of the triceps brachii muscle, GR - graft repair, FCR-IP - the transfer of the branch of the median nerve for the flexor carpi radialis muscle to the posterior interosseus nerve, FDS-ECRB - the transfer of the branch of the median nerve for the flexor digitorum superficialis muscle to the branch of the radial nerve for the extensor carpi radialis brevis muscle, PT-ECRB - the transfer of the tendon of the pronator teres muscle to the tendon of the extensor carpi radialis brevis muscle, PQ-MFU -the transfer of the terminal branch of the anterior interosseus nerve to the motor fascicle of the ulnar nerve, LCN-SFU-the transfer of the lateral cutaneous nerve of the forearm to the sensory fascicle of the ulnar nerve; ***Results:*** EF - elbow flexion, SU - elbow supination, EX - elbow extension, RoM - range of motion, FRoM - full range of motion, *Peripheral Nerve Surgery Quality of Life questionnaire:* PrePNSQoL - preoperative PNSQoL, PostPNSQoL - postoperative PNSQoL, *Medical Research Council muscle strength grading scale:* M0 - MRC M0, M1 - MRC M1, M2 - MRC M2, M3 - MRC M3, M4 - MRC M4, M5 - MRC M5.
