# Supplementary material for: Quality of life in patients following distal nerve transfers for the restoration of elbow flexion and extension- single center experience
Source: Brain Spine. 2026 Jul 2;6:106156. doi: 10.1016/j.bas.2026.106156 (PMC13348191; doi:10.1016/j.bas.2026.106156)
Supplement: Multimedia component 2 [file mmc2.docx]

***Apendix B.*** Peripheral Nerve Surgery Quality of Life (PNSQoL) questionnaire.

Peripheral Nerve Surgery Quality of Life (PNSQoL) questionnaire is an original questionnaire designed by the authors (Clinic for Neurosurgery, University Clinical Centre of Serbia) to improve quality of life (QoL) assessment in patients undergoing upper and/or lower extremity peripheral nerve surgery (PNS).

The questionnaire provides a structured evaluation of a patient's ability to perform daily activities, social interactions, professional engagement, and overall satisfaction with their condition before and/or after surgery. Questions 13, 14, and 15 explicitly differentiate between a patient's state before or after surgery.

The score ranges from 0-80, with higher scores indicating better QoL. For descriptive interpretation, postoperative scores were additionally categorized as poor (0–40), fair (40–50), good (50–60), very good (60–70), and excellent (70–80). All answers must be provided for the score to be valid.

The patients in this study completed the Serbian version of the PNSQoL questionnaire.

**PNSQoL questionnaire (English version)**

1. **How would you rate your ability to independently maintain personal hygiene (washing your face, brushing your teeth, showering, combing, drying your hair)?**
   1. unable
   2. able with extreme difficulty
   3. able with great difficulty
   4. able with moderate difficulty
   5. able with difficulty
   6. able without difficulty
2. **How would you rate your ability to dress yourself?**
   1. unable
   2. able with extreme difficulty
   3. able with great difficulty
   4. able with moderate difficulty
   5. able with difficulty
   6. able without difficulty
3. **How would you rate your ability to independently pick up a glass from a table and drink the content?**
   1. unable
   2. able with extreme difficulty
   3. able with great difficulty
   4. able with moderate difficulty
   5. able with difficulty
   6. able without difficulty
4. **How would you rate your ability to independently eat food someone else prepared and served on the table (use of cutlery)?**
   1. unable
   2. able with extreme difficulty
   3. able with great difficulty
   4. able with moderate difficulty
   5. able with difficulty
   6. able without difficulty
5. **How would you rate your ability to independently do housework (making the bed, folding clothes, cooking, vacuuming, throwing the trash away, gardening)?**
   1. unable
   2. able with extreme difficulty
   3. able with great difficulty
   4. able with moderate difficulty
   5. able with difficulty
   6. able without difficulty
6. **How would you rate your ability to go grocery shopping on your own?**
   1. unable
   2. able with extreme difficulty
   3. able with great difficulty
   4. able with moderate difficulty
   5. able with difficulty
   6. able without difficulty
7. **How would you rate your ability to participate in recreational activities (walking, running, swimming, playing football, playing basketball)?**
   1. unable
   2. able with extreme difficulty
   3. able with great difficulty
   4. able with moderate difficulty
   5. able with difficulty
   6. able without difficulty
8. **How would you rate your ability to continue with your work-related activities (going to school, going to university, going to work)?**
   1. unable
   2. able with extreme difficulty
   3. able with great difficulty
   4. able with moderate difficulty
   5. able with difficulty
   6. able without difficulty
9. **Do you have sleep problems because of the current condition of your extremity?**
   1. Everyday
   2. Almost every day
   3. Often
   4. Sometimes
   5. Rarely
   6. Never
10. **In the past month, have you experienced people from your surroundings feeling pity for you because of the current condition of your extremity?**
    1. Everyday
    2. Almost every day
    3. Often
    4. Sometimes
    5. Rarely
    6. Never
11. **In the past month, have you experienced humiliation or discrimination because of the current condition of your extremity?**
    1. Everyday
    2. Almost every day
    3. Often
    4. Sometimes
    5. Rarely
    6. Never
12. **In the past month, has your social life been influenced by the current condition of your extremity (relationship with your family, friends, colleagues)?**
    1. Everyday
    2. Almost everyday
    3. Often
    4. Sometimes
    5. Rarely
    6. Never
13. **In the past month, has the current condition of your extremity limited your everyday activities (taking care of yourself, taking care of your family, taking care of your household, going to school, going to university, going to work, doing work-related tasks)?**
    1. Everyday
    2. Almost every day
    3. Often
    4. Sometimes
    5. Rarely
    6. Never
14. **Are you satisfied with the current condition of your extremity compared to its condition before the symptom symptom/injury/disorder/disease onset or its condition before the surgery?**
    1. Very unsatisfied
    2. Moderately unsatisfied
    3. Slightly unsatisfied
    4. Slightly satisfied
    5. Moderately satisfied
    6. Very satisfied
15. **Are you satisfied with your current social life compared to your social life before the symptom/injury/disorder/disease onset or before the surgery?**
    1. Very unsatisfied
    2. Moderately unsatisfied
    3. Slightly unsatisfied
    4. Slightly satisfied
    5. Moderately satisfied
    6. Very satisfied
16. **Are you satisfied with your current professional life compared to your professional life before the symptom/injury/disorder/disease or before the surgery?**
    1. Very unsatisfied
    2. Moderately unsatisfied
    3. Slightly unsatisfied
    4. Slightly satisfied
    5. Moderately satisfied
    6. Very satisfied

**PNSQoL questionnaire (Serbian version)**

1. **Kako biste ocenili svoju sposobnost da samostalno održavate ličnu higijenu (umivanje, pranje zuba, tuširanje, češljanje, feniranje)?**
   1. Nesposoban/na
   2. Sposoban/na sa veoma velikim teškoćama
   3. Sposoban/na sa velikim teškoćama
   4. Sposoban/na a sa umerenim teškoćama
   5. Sposoban/na sa lakim teškoćama
   6. Sposoban/na bez teškoća
2. **Kako biste ocenili svoju sposobnost da se samostalno obučete?**
   1. Nesposoban/na
   2. Sposoban/na sa veoma velikim teškoćama
   3. Sposoban/na sa velikim teškoćama
   4. Sposoban/na sa umerenim teškoćama
   5. Sposoban/na sa lakim teškoćama
   6. Sposoban/na bez teškoća
3. **Kako biste ocenili svoju sposobnost da samostalno uzmete, sa stola, čašu vode i popijete je?**
   1. Nesposoban/na
   2. Sposoban/na sa veoma velikim teškoćama
   3. Sposoban/na sa velikim teškoćama
   4. Sposoban/na sa umerenim teškoćama
   5. Sposoban/na sa lakim teškoćama
   6. Sposoban/na bez teškoća
4. **Kako biste ocenili svoju sposobnost da samostalno jedete hranu koju vam je neko drugi pripremio i postavio na sto (korišćenje kašike, viljuške i noža)?**
   1. Nesposoban
   2. Sposoban/na sa veoma velikim teškoćama
   3. Sposoban/na sa velikim teškoćama
   4. Sposoban/na sa umerenim teškoćama
   5. Sposoban/na sa lakim teškoćama
   6. Sposoban/na bez teškoća
5. **Kako biste ocenili svoju sposobnost da samostalno obavljate kućne poslove (raspremanje kreveta, slaganje odeće, kuvanje, usisavanje, bacanje smeća, sredjivanje dvorišta)?**
   1. Nesposoban
   2. Sposoban/na sa veoma velikim teškoćama
   3. Sposoban/na sa velikim teškoćama
   4. Sposoban/na sa umerenim teškoćama
   5. Sposoban/na sa lakim teškoćama
   6. Sposoban/na bez teškoća
6. **Kako biste ocenili svoju sposobnost samostalnog odlaska u prodavnicu radi kupovine namirnica za kuću?**
   1. Nesposoban/na
   2. Sposoban/na sa veoma velikim teškoćama
   3. Sposoban/na sa velikim teškoćama
   4. Sposoban/na sa umerenim teškoćama
   5. Sposoban/na sa lakim teškoćama
   6. Sposoban/na bez teškoća
7. **Kako biste ocenili svoju sposobnost učestvovanja u rekreativnim aktivnostima (šetnja, trčanje, plivanje, fudbal, basket)?**
   1. Nesposoban/na
   2. Sposoban/na sa veoma velikim teškoćama
   3. Sposoban/na sa velikim teškoćama
   4. Sposoban/na sa umerenim teškoćama
   5. Sposoban/na sa lakim teškoćama
   6. Sposoban/na bez teškoća
8. **Kako biste ocenili svoju sposobnost da nastavite sa svojim radnim aktivnostima (učenje škole, studiranje, rad na svom radnom mestu)?**
   1. Nesposoban/na
   2. Sposoban/na sa veoma velikim teškoćama
   3. Sposoban/na sa velikim teškoćama
   4. Sposoban/na sa umerenim teškoćama
   5. Sposoban/na sa lakim teškoćama
   6. Sposoban/na bez teškoća
9. **Da li zbog trenutnog stanja vašeg ekstremiteta imate probleme sa spavanjem?**
   1. Svakodnevno
   2. Skoro svakodnevno
   3. Često
   4. Ponekad
   5. Retko
   6. Nikad
10. **Da li ste zbog trenutnog stanja vašeg ekstremiteta osetili sažaljenje ljudi iz vaše okoline u poslednjih mesec dana?**
    1. Svakodnevno
    2. Skoro svakodnevno
    3. Često
    4. Ponekad
    5. Retko
    6. Nikad
11. **Da li ste zbog trenutnog stanja vašeg ekstremiteta doživeli poniženje ili diskriminaciju u poslednjih mesec dana?**
    1. Svakodnevno
    2. Skoro svakodnevno
    3. Često
    4. Ponekad
    5. Retko
    6. Nikad
12. **Da li je stanje vašeg ekstremiteta u poslednjih mesec dana uticalo na vaš socijalni život (odnos sa porodicom, prijateljima, kolegama)?**
    1. Svakodnevno
    2. Skoro svakodnevno
    3. Često
    4. Ponekad
    5. Retko
    6. Nikad
13. **Da li vas je stanje ekstremiteta u poslednjih mesec dana ograničavalo u izvođenju svakodnevnih radnih aktivnosti (briga o sebi, briga o porodici, briga o kući, odlazak u školu, odlazak na fakultet, odlazak na posao, izvođenje predviđenih radnih zadataka)?**
    1. Svakodnevno
    2. Skoro svakodnevno
    3. Često
    4. Ponekad
    5. Retko
    6. Nikad
14. **Da li ste zadovoljni trenutnim stanjem vašeg ekstremiteta u odnosu na stanje pre pojave simptoma/povrede/bolesti, odnosno na stanje pre operacije?**
    1. Veoma nezadovoljan/na
    2. Umereno nezadovoljan/na
    3. Diskretno nezadovoljan/na
    4. Diskretno zadovoljan/na
    5. Umereno zadovoljan/na
    6. Veoma zadovoljan/na
15. **Da li ste zadovoljni vašim trenutnim socijalnim životom u odnosu na stanje pre pojave simptoma/povrede/bolesti, odnosno na stanje pre operacije?**
    1. Veoma nezadovoljan/na
    2. Umereno nezadovoljan/na
    3. Diskretno nezadovoljan/na
    4. Diskretno zadovoljan/na
    5. Umereno zadovoljan/na
    6. Veoma zadovoljan/na
16. **Da li ste zadovoljni vašim trenutnim profesionalnim životom u odnosu na stanje pre pre pojave simptoma/povrede/poremećaja/bolesti, odnosno na stanje pre operacije?**
    1. Veoma nezadovoljan/na
    2. Umereno nezadovoljan/na
    3. Diskretno nezadovoljan/na
    4. Diskretno zadovoljan/na
    5. Umereno zadovoljan/na
    6. Veoma zadovoljan/na
